# Perceptions and Experiences with Flavored Non-Menthol Tobacco Products: A Systematic Review of Qualitative Studies

**DOI:** 10.3390/ijerph14040338

**Published:** 2017-03-23

**Authors:** Sarah D. Kowitt, Clare Meernik, Hannah M. Baker, Amira Osman, Li-Ling Huang, Adam O. Goldstein

**Affiliations:** 1 Department of Health Behavior, Gillings School of Global Public Health, University of North Carolina at Chapel Hill, Chapel Hill, NC 27599, USA; 2 Lineberger Comprehensive Cancer Center, University of North Carolina at Chapel Hill, 101 Manning Dr, Chapel Hill, NC 27514, USA; hmbaker2@unc.edu (H.M.B); amirao@email.unc.edu (A.O.); liling_huang@med.unc.edu (L.-L.H.); adam_goldstein@med.unc.edu (A.O.G.); 3 Department of Family Medicine, University of North Carolina at Chapel Hill, 590 Manning Drive, Chapel Hill, NC 27599, USA; cmeernik@email.unc.edu

**Keywords:** other tobacco products, tobacco control, flavored tobacco products, regulation

## Abstract

Although a few countries have banned flavored cigarettes (except menthol), flavors in most tobacco products remain unregulated across the globe. We conducted a systematic review of qualitative studies examining perceptions of and experiences with flavored non-menthol tobacco products. Of 20 studies on flavored tobacco products included in our qualitative systematic review, 10 examined hookah, six examined e-cigarettes, two examined little cigars and cigarillos (LCCs), and three examined other tobacco products, including cigarettes. The majority of studies, regardless of product type, reported positive perceptions of flavored tobacco products, particularly among young adults and adolescents. In six studies that assessed perceptions of harm (including hookah, LCCs, and other flavored tobacco products), participants believed flavored tobacco products to be less harmful than cigarettes. In studies that examined the role of flavors in experimentation and/or initiation (including three studies on e-cigarettes, one hookah study and one LCC study), participants mentioned flavors as specifically leading to their experimentation and/or initiation of flavored tobacco products. Given that many countries have not yet banned flavors in tobacco products, these findings add to existing research on why individuals use flavored tobacco products and how they perceive harm in flavored tobacco products, providing further support for banning non-menthol flavors in most tobacco products.

## 1. Introduction

Flavored tobacco product use among youth and young adults is high, with prevalence rates for current use (i.e., past 30 days) of any flavored tobacco product ranging from 12% among youth to 18.5% among young adults in the United States (U.S.) [1,2]. When examined by product type for young adults, use of any flavored tobacco product ranged from 3% for cigarettes (excluding menthol), to 13% for e-cigarettes, 35% for little cigars/cigarillos or bidis, and 50% for hookah. Additionally, many adolescents initiate tobacco use with flavored products [3], and flavors are one of the main reasons why youth continue to use tobacco products [3]. The 2009 Family Smoking Prevention and Tobacco Control Act authorized the U.S. Food and Drug Administration (FDA) to ban cigarettes with characterizing flavors (except for menthol) [4], but flavors in other non-cigarette tobacco products remain legal [5]. While other countries have also banned flavors in cigarettes (e.g., countries in the European Union), most countries have not enacted such bans, with very few regulating flavors in non-cigarette tobacco products [6,7].

Moreover, while comprehensive reviews have examined the public health impact of menthol tobacco products [8,9,10,11,12,13], only two systematic reviews have examined non-menthol flavored tobacco products. Feirman, et al., examined the use of and attitudes towards non-menthol flavored tobacco products in quantitative (*n* = 26) and qualitative (*n* = 6) studies published in the U.S., prior to September 2013 [14]. Huang et al. examined quantitative data (*n* = 40) on the role of flavors on attitudes, perceptions, intentions, initiation, and cessation of non-menthol flavored tobacco products in U.S. and non-U.S. studies published through April 2016 [15]. Qualitative research serves a complementary yet separate purpose from quantitative research; researchers often use qualitative data to gain a better understanding of the context of a problem, describe complex relationships, and explore new topics [16]. To extend these previous two reviews and further explore individuals’ perceptions of and experiences with flavored tobacco products—specifically why individuals use flavored tobacco products and how they perceive them—we conducted a systematic review of qualitative studies of non-menthol flavored tobacco products through April 2016, including international and more recent qualitative studies not previously reported [14].

## 2. Materials and Methods

### 2.1. Inclusion/Exclusion Criteria

Studies that included populations of any age, race, sex, ethnicity, or country and described individuals’ experiences with or perceptions toward non-menthol flavored tobacco products were included. We excluded articles that were not qualitative; were not English-language; were not peer-reviewed; did not contain original data about flavored tobacco products; did not address the specific impact of flavors on tobacco product perceptions and use behaviors; or limited findings to menthol flavored tobacco products only.

### 2.2. Data Sources and Study Selection

**Figure 1:**
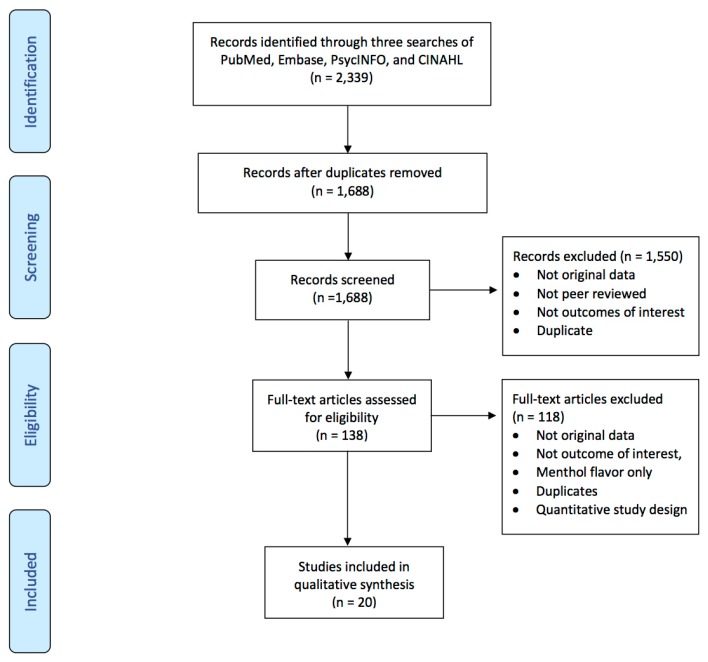
PRISMA flow diagram of article identification, screening and selection.

Four databases (PuBMed, Embase, PsycINFO, CINAHL) were used for our search. One author (Hannah M. Baker) searched all of the databases using cognates of “tobacco products” and “flavor” and two authors (Clare Meernik and Hannah M. Baker) conducted a manual search of the reference lists in each included article (see Figure 1). Searches were conducted in three waves: March 2015, September 2015, and April 2016. There were no date restrictions regarding when articles were published. After duplicates were removed, a total of 1688 articles were found. After reviewing titles and abstracts, 138 full-text articles were assessed for eligibility. A total of 20 articles were included in the final analysis. For a full description of our search strategy and study selection, please refer to Huang et al. [15].

Of the 20 studies included, only two were included in any previous review of use of and attitudes toward flavored tobacco products in the United States; 18 studies were not included in any prior flavor systematic reviews. We excluded four qualitative articles from Feirman et al.’s review since they did not specifically address the impact of flavors on perceptions or behavioral outcomes (*n* = 3) or the article type was a dissertation (not a peer reviewed study) (*n* = 1).

### 2.3. Data Extraction

Two authors (Clare Meernik and Hannah M. Baker) independently extracted data, which included study aim, type of flavored tobacco product, characteristics of study populations and study design, and main results and findings related to the impact of flavors in tobacco products. As findings may differ by type of flavored tobacco product, we synthesized results by type of product. We used the validated Quality Assessment Tool (QATSDD) to assess the quality of studies, [17]. The tool includes 14 criteria that apply to qualitative studies (e.g., explicit theoretical framework; description of procedure for data collection; assessment of reliability of analytic process). Two authors (Clare Meernik and Hannah M. Baker) rated each study on a 4-point scale ranging from “0 = did not address criteria at all” to “3 = completely addressed criteria”. Scores were then summed (range 0–42), with higher scores indicating higher quality. Although we did not use these criteria to rank or exclude studies, they provided information which we used in interpreting findings.

## 3. Results

### 3.1. Study Characteristics

Half of the 20 studies were conducted in the U.S. (*n* = 10); the remaining studies were conducted in Scotland, the United Kingdom (UK), the Middle East, Canada, and Malaysia (see Table 1). All of the studies were published in 2009 or after. Half of the studies used one-on-one interviews to collect data (*n* = 9), while the remainder used focus groups (*n* = 8) or a combination of focus groups and one-on-one interviews (*n* = 3). The majority of study participants were young adults between the ages of 18–30, although four studies included adolescents and four studies included adults above the age of 30. In addition, studies included mainly users of tobacco products (*n* = 11) or both users and non-users of tobacco products (*n* = 8). Quality scores for included studies ranged from 15 to 38, with a mean quality score of 27.6 (Standard Deviation: 5.9) and a median score of 27.

### 3.2. Results by Type of Tobacco Product

Of 20 studies on flavored tobacco products included in our qualitative systematic review, 10 examined hookah, six examined e-cigarettes, two examined little cigars and cigarillos (LCCs), and three examined other tobacco products, including cigarettes. Results, organized by type of tobacco product, can be seen in Table 2.

#### 3.2.1. Hookah

Ten studies examined flavors in waterpipe or hookah (hereafter referred to as hookah), most of which were studies from Western countries (Scotland, Canada, UK, U.S.) (see Table 2) [18,19,20,21,22,23,24,25,26,27]*.* In all ten studies, participants spoke about flavors as appealing and/or tasty; in five of the studies, participants also spoke positively about the widespread availability of different flavors and the ability to mix flavors [18,22,24,26,27].

In four studies from Canada, the UK, and the U.S., young adults reported that they used hookah specifically because of the flavors and noted that they would not use other tobacco products, such as cigarettes or piped tobacco [19,22,25,26]. As one young adult user said, “Lots of people I know smoke shisha and don’t smoke cigarettes because they don’t like the taste of it, whereas shisha tastes really nice” [22]. In part, these participants noted the appealing taste of flavors; they also reported believing that flavored hookah was less harmful than cigarettes, as reported in three studies from Canada, the U.S., and the UK [19,20,25]. As one young adult user said, “Fruit flavor makes it less harmful. I don’t believe it’s as harmful as cigarettes. Everyone seems to believe this, that it’s less toxic. I know students who smoke shisha but wouldn’t smoke cigarettes. If offered a cigarette, they’d turn it down” [25].

In two studies from Canada and Lebanon, adult users and non-users of hookah said that flavors were a reason why younger people used hookah [21,24], and in one study among young adults in Canada, flavors were mentioned as the reason for initiating use of hookah: “I think it had to do with the flavor that got me to smoke it. It has a lot of variety” [20]. In one study among young adults in the UK, users described being addicted to hookah, in part because of flavors [22]. Lastly, in a study with 27 adults in Canada, most participants felt that there should be additional restrictions on flavored hookah [21].

#### 3.2.2. E-Cigarettes

Six studies examined flavors in e-cigarettes, e-hookahs, e-shisha, and vape pens (hereafter referred to as e-cigarettes) [22,28,29,30,31,32]. All studies discussed how flavors contributed to the appeal of e-cigarettes. Two studies from the U.S. also mentioned how flavors allowed e-cigarette users to be more social, for instance, by serving as a conversation starter or by allowing people to share flavors in social settings [28,31].

In three studies with young adults and adolescents from the U.S. and Scotland, participants mentioned how flavors contributed to their initiation, experimentation, and/or continued use of e-cigarettes or other tobacco products [28,30,32]. Young adult users of hookah in the UK described the belief that use of e-cigarettes (especially tobacco flavored) could lead to use of other tobacco products, stating, “Non-smokers could then be encouraged to smoke and try tobacco, like actual cigarettes and tobacco flavored shisha” [22]. Additionally, in a study in Scotland, some adolescents felt that e-cigarettes should be regulated because these devices could lead younger generations to start smoking [32]. Conversely, in one U.S. study, two e-cigarette users (out of 50 total participants) noted that they felt that flavors played an instrumental (positive) role in cigarette smoking cessation, stating “If I don’t like the flavor, I’m going to smoke a cigarette in a weird way, because it's not satisfying. It’s like I'm a slave to nicotine, but if you find a flavor that you like, you're more inclined to be like, “This is sufficient. I don’t want (a cigarette)” [29].

#### 3.2.3. Little Cigars and Cigarillos (LCCs)

Two studies examined flavors in LCCs among U.S. young adult users of cigarettes and/or LCCs [33,34]. In one study, participants perceived flavored LCCs as less harmful than regular cigarettes due to the packaging, colors, and taste of the flavors [33]. For example, one participant said, “Also the flavors they put the fruits there...people might assume that it’s okay it’s got fruits on it, cause this one’s even got a picture of a fruit all cut up and it looks good. So, they’re associating it with the natural” [33]. In another study, participants noted the appeal of flavored LCCs and described how flavors and their variety led to experimentation and initiation of LCCs [34]. For example, one participant said, “And I was like it (LCC) smells good, so it made me want to hit it. But I’m like I don’t like the taste of this. So, then I started of course experimenting with different ones” [34]. In this same study, participants also noted that flavored LCCs affected smoking cessation. Specifically, one participant discussed a friend who thought he could quit smoking cigarettes by using flavored LCCs since they take longer to burn. However, at the end of the day, the friend ended up smoking more flavored LCCs than originally intended and not being able to quit cigarettes. Thus, in this case, use of flavored LCCs actually perpetuated tobacco use [34].

#### 3.2.4. Other Tobacco Products 

Three studies examined flavors in other tobacco products, which included dissolvables, snus, cigarettes, and other unspecified products [35,36,37]. In these studies, young adults in the U.S. described the appeal of dissolvable tobacco products [36] and the belief that flavored tobacco products made people think these products were safer than cigarettes given their more tolerable odor [37]. Additionally, in one cigarette study where adolescents and young adults in Scotland were shown different colored cigarettes (standard, pink, brown), participants perceived the pink cigarette as more appealing and less harmful because it looked more like “candy sticks” and its color indicated a nicer taste, regardless of the actual flavor of the product [35].

## 4. Discussion

This is the first systematic review examining qualitative studies in the U.S. and internationally of non-menthol flavored tobacco products. Our review extends research by Feirman et al. [14], by including international studies, incorporating more studies on non-cigarette products, such as hookah, examining harm perceptions in addition to appeal, and reviewing recent literature published in the last three years.

Supporting previous quantitative research on the appeal of flavored tobacco products [1,2,3], we found that nearly all 20 studies included in our review reported positive perceptions of flavored tobacco products. Regardless of product type, common reasons for the appeal of flavored tobacco products included the availability and novelty of flavors; the attractiveness of product packaging and marketing of flavors; the associations between certain flavors and relaxation; the tastiness of flavors; the ability to mix flavors (especially for e-cigarettes); and the social aspect of sharing flavors. Moreover, all but four of these studies focused on adolescents and young adults, and all but one study focused on non-cigarette flavored tobacco products, such as hookah and e-cigarettes, suggesting that flavored tobacco products (other than cigarettes) are widely appealing to adolescents and young adults.

Given the favorability of flavored non-menthol tobacco products, the reasons why individuals use these products, and the appeal of these products to youth and young adults, our findings extend support for banning non-menthol flavors in tobacco products. A growing number of studies have established the harmfulness of non-cigarette tobacco products, including hookah [38], cigars [39] and e-cigarettes [40,41,42,43], particularly for adolescents. These products contain nicotine, which has been shown to be harmful for developing brains of adolescents [44], and early exposure to nicotine may lead to future tobacco product use. For instance, longitudinal research has shown that e-cigarette use in adolescence is associated with future tobacco product use, including cigarettes [45,46,47,48]. Thus, our findings point to regulation of all flavored tobacco products, including e-cigarettes, since they are heavily marketed to youth and may be detrimental to brain development and for future tobacco product use. Furthermore, in two studies included in our review (one involving adults’ opinions of hookah; one involving adolescents’ opinions of e-cigarettes), individuals reported favorable attitudes toward regulation of flavored hookah and e-cigarettes as a way to deter youth initiation of tobacco [21,32]. These findings may be useful to tobacco regulatory agencies as further evidence regarding the importance of regulation of flavored non-menthol tobacco products, as well as the acceptability of potential regulation.

Most concerning in our review was the fact that in six studies that assessed harm perceptions (examining hookah, LCCs, and other flavored tobacco products), participants mentioned flavors as a reason why they perceive certain tobacco products as less harmful than cigarettes [19,20,25,33,35,37]. Prior systematic reviews from Huang et al. [15] and Feirman et al. [14] identified several quantitative studies illustrating associations between flavor descriptors and reduced harm perceptions. Our study extends these findings by highlighting potential pathways though which these associations occur. For example, product packaging including colors and flavor descriptors (e.g., fruit flavors and naturalness), and the sweet fruity smells of flavored tobacco products appear to act as implicit modified risk claims that lead users to perceive flavored products as less harmful than cigarettes. Future research should investigate ways in which flavors alter risk perception of tobacco use. Public health interventions and education campaigns could target beliefs associated with flavors to increase risk perceptions of flavored tobacco products and decrease initiation and/or consumption of such products. Moreover, given these patterns across different flavored tobacco products, regulation of *all* types of flavored tobacco products (rather than some specific types) may be important.

Participants across studies mentioned how flavors led to their experimentation and/or initiation of flavored tobacco products, consistent with previous quantitative research describing the role of flavors in smoking initiation [1,2,3]. For instance, in a large nationally representative survey in the U.S., researchers found that the majority of youth ever-tobacco users (81%) reported that the first product they used was flavored [3]. The relationship between flavored tobacco product use and cigarette smoking cessation is less consistent. In our review, two studies (one on e-cigarettes and one on LCCs) provided participants’ perspectives on how flavors may affect cigarette use. Specifically, participants mentioned that e-cigarette flavors could aid smoking cessation (if a participant liked a flavored e-cigarette, they would not feel the need to smoke cigarettes) [29] and that flavored LCCs may deter cigarette smoking cessation (by perpetuating tobacco use) [34].

Flavors are often viewed as a major reason for young people starting out using tobacco products (often with lower nicotine) and moving up to stronger products (with higher nicotine content) over time [1]. Interestingly we found that in four hookah studies, participants noted that they would use flavored hookah, but not other tobacco products, such as cigarettes [19,22,25,26]. On the other hand, in two e-cigarette studies, participants noted that use of flavored e-cigarettes could lead to use of other tobacco products, such as hookah or cigarettes [22,32]. These findings suggest that flavored hookah or e-cigarettes may appeal to different segments of the population than cigarettes. Given associations between initiation of flavored tobacco products in youth or young adulthood and established tobacco use later in life [1], our results suggest the potential of flavors to introduce youth to other tobacco products, but that these patterns may differ by type of product first used. Future longitudinal research could examine whether the type of flavored tobacco products plays a differential role in tobacco use patterns throughout the life course.

### Limitations

Our study had several limitations. First, our review is limited to studies published in peer-reviewed journals; other relevant information may have been reported in reports, dissertations, or the grey literature, which would not be captured in this review. Second, no published articles examined the role of flavors in attitudes, perceptions, or experiences with smokeless tobacco products. Third, while we reported quotes from participants and described the context of the quotes, we were not able to assess the extent to which beliefs were held or stated by participants in the studies. In other words, we were unable to assess the prevalence of specific beliefs. Fourth, many studies did not state qualities of participants giving quotes (e.g., age, smoking status, etc.), which limits our ability to differentiate themes or results by types of participants. Fifth, most of the studies did not include enough primary data (e.g., participant quotes) to use a theoretical framework to extract themes form the body of research we reviewed. As a result, we were unable to employ a theoretically grounded approach to evaluate these studies. Sixth, while we assessed the quality of studies included in this review using a validated tool, there are no published thresholds for what constitutes low, medium, or high quality. As a result, we did not use criteria to rank or exclude studies. Instead, since 95% of the studies included in our review met at least 50% of the recommended criteria in our quality assessment tool (i.e., the highest score of 42), we retained all eligible studies and did not exclude articles based on quality information. Last, our review focused on perceptions and experiences with non-menthol flavored tobacco products. While previous reviews have examined the public health impact of menthol [8,9,10,11,12,13], future research could explore the role of menthol in attitudinal and behavioral outcomes among diverse populations and various tobacco products.

## 5. Conclusions

Our review extends findings from quantitative studies on flavored tobacco products and provide insights into perceptions of and experiences with flavored tobacco products. More specifically, we found that in nearly all 20 studies included in our review, participants reported positive perceptions of flavored tobacco products and mentioned how flavors led to their experimentation and/or initiation of flavored tobacco products. Moreover, all but four of these studies focused on adolescents and young adults, and all but one study focused on non-cigarette flavored tobacco products, such as hookah and e-cigarettes, suggesting that flavored tobacco products (other than cigarettes) are widely appealing to this population. Our findings provide additional support to ban non-menthol flavors in tobacco products in the US and internationally, especially to curb tobacco use among youth.

## Figures and Tables

**Table 1:** Characteristics of included studies, *n* = 20.

Study Characteristic	*n* or Mean (SD)
**Location of study**	
U.S. studies	10
Non-U.S. studies	10
**Year of publication**	
Between 2009 and 2012	6
Between 2013 and 2016	14
**Methodology used**	
Focus groups	8
One-on-one interviews	9
Both	3
**Age of participants ^1^**	
Adolescents (<18)	5
Young adults (18–30)	16
Adults (18+)	4
**Tobacco Use Status of Participants**	
Users	11
Non-users	0
Both users and non-users	8
Unspecified	1
Cigarette	1
E-cigarette	6
Hookah, waterpipe	10
Little cigar, cigarillo, cigar	2
Various non-cigarette products	2
**Sample size**	
Greater than or equal to 50 participants	12
Less than 50 participants	8
**Quality of studies ^2^**	
Mean (range: 15–38)	27.6 (SD: 5.9)

**^1^** Categories are not mutually exclusive. **^2^** Quality scores range from 0–42.

**Table 2:** Main study findings, organized by type of tobacco product.

Study ID	Product	Study Setting & Population	Sample Size	Quality Score	Main Findings and Illustrative Quotes
Afifi, 2013 [18]	Hookah	Middle Eastern countries (Lebanon, Syria, Palestine, Egypt) Young adults, adults Users and non-users of hookah	81 focus group discussion (8–10 people per group), 38 one-on-one interviews	38	Participants described a variety of sensory characteristics (i.e., taste and smell of fruit flavored tobacco) that contributed to hookah use. Participants also spoke of the range of hookah tobacco flavors (i.e., fruit, mint, lemon, chocolate, energy drinks) and the novelty of flavors.
Griffiths 2011 [19]	Hookah	U.S. Young adults Users of hookah (current and past)	20 one-on-one interviews	23	Participants described the appeal of fruit-flavored hookah and reported that the fruit flavors disguise the true risks of smoking hookah (compared to cigarettes). ○ “I mean, it’s mostly flavoring that you get from it, like cherry. So I don’t think there’s bad stuff in that, I mean we eat strawberry and cherry flavor stuff in food, and that’s not bad, so I don’t think sheesha is bad.” Participants also reported that the flavors in hookah are similar to foods and denied that hookah contained toxins or is addictive. ○ “I know it’s a lot better than smoking cigarettes, which I don’t smoke cigarettes or anything else.... I like the flavored sheesha and you can’t get high off of that. There’s nothing in it, it’s mostly just flavoring with natural herbs and probably a little bit of tobacco just so that it burns with the coals. So, no, I don’t think it’s risky at all.”
Hammal, 2016 [20]	Hookah	Canada Young adults Users of hookah	16 focus groups (75 participants)	24	Participants believed that the product packaging (fruits and bright colors) were appealing and gave off the impression that hookah was safer than cigarettes. ○ “Right now the packages are all bright colors and they attract your eyes because when you see them there is strawberry and kiwi and all the nice flavors so I believe putting pictures on it is a good way, it does veer off some people” Participants also discussed how flavors led them to initiate hookah use. ○ “I think it had to do with the flavor that got me to smoke it. It has a lot of variety.”
Hammal, 2016 [21]	Hookah	Canada Adults Unspecified whether users or non-users were included	27 one-on-one interviews	15	Participants mentioned that they thought flavors were a reason why younger smokers started using hookah. ○ “The taste and all kinds of fruits, all kinds of flavors and they like to fit, especially the new generation” Most participants also felt that there should be additional restrictions on flavored products.
Kotecha, 2016 * [22]	Hookah	UK Young adults Users of hookah	16 one-on-one interviews	33	Participants described hookah flavors and the ability to mix flavors as appealing and able to mask the pungent taste of tobacco. ○ “Lots of people I know smoke shisha and don’t smoke cigarettes because they don’t like the taste of it, whereas shisha tastes really nice” Participants also described being addicted to hookah, in part because of the flavors. ○ “I’m addicted to it...I just like the whole flavor, the whole mechanism of doing it, the process of doing it really”
Jawad, 2013 [23]	Hookah	UK Young adults Users of hookah	7 focus groups (32 participants)	24	Participants described the appeal of flavors (taste, smell, sight).
Nakkash, 2011 [24]	Hookah	Lebanon Adults Users and non-users of hookah	25 focus groups (8–10 people per group) and 9 one-on-one interviews	31	Participants (especially women, younger participants, and smokers) described how innovations in tobacco flavors contributed to increased hookah use and motivated hookah initiation. Participants also noted widespread advertising of flavors. ○ “The companies are making use of it commercially, once they introduce the grape flavor to the market, people would then want to try it out, once they introduce the peach flavor, again people would want to try it out... I mean I think if they had only stopped with the apple flavor, maybe a lot of people would have had gave up waterpipe smoking.”
Roskin, 2009 [25]	Hookah	England and Canada Young adults Users of hookah	12 one-on-one interviews	27	Flavors were noted as primary explanation for hookah’s relaxing appeal. ○ “The strong flavor and strong smoke are great. I can do smoke rings and impress the ladies. There would be no point in smoking if it wasn't flavored” One participant directly equated flavors with candy. ○ “The fruit flavor makes it like a candy, it's a silly assumption to make, but it's my assumption” Participants said that fruit flavors make hookah less harmful and less toxic than cigarettes. ○ “Fruit flavor makes it less harmful. I don’t believe it’s as harmful as cigarettes. Everyone seems to believe this, that it’s less toxic. I know students who smoke shisha but wouldn’t smoke cigarettes. If offered a cigarette, they’d turn it down.”
Sharma, 2014 [26]	Hookah	U.S. Young adults Users of hookah	49 one-on-one interviews	33	One of the most common reasons for smoking hookah, as noted by participants, was the availability of multiple tobacco flavors. Participants also described that they would be unlikely to use hookah if it was not flavored. ○ “It tastes good and there are so many different varieties. I don’t know if I will smoke waterpipe if it was not flavored. I tried piped tobacco once, it was harsh.”
Yen, 2012 [27]	Hookah	Malaysia Adolescents Users of hookah	5 one-on-one interviews	16	Many participants noted that they use hookah because of the flavors, the ability to mix flavors, and the taste of hookah (in comparison to cigarettes).
Cheney, 2016 [28]	E-cigarette	U.S. Young adults Users of e-cigarettes	30 one-on-one interviews	34	Participants described how flavors allowed them to be social with other e-cigarette users. ○ “I feel like if somebody walked by me and I was vaping they’d be like oh what kind of flavor is that? Nobody walks by you while you’re smoking and says oh what brand are you smoking?” Participants also noted how flavors contributed to their initial attraction and use of e-cigarettes, experimentation with e-cigarettes (by trying a friend’s e-cigarette), and continued use of e-cigarettes (since they were so many flavors). ○ “I started vaping and it’s that flavor it’s that delicious flavor that you get when you get to blow out smoke and it’s like fruity and the only time that you ever get a fruity flavor in a cigarette is when you smoke those little cigars blah.” Participants also discussed how flavors were fun and allowed them to be creative because of mixing.
Cooper, 2016 [29]	E-cigarette	U.S. Young adults and Adults Users of e-cigarettes	50 one-on-one interviews	26	Participants described trying a wide variety of flavors and mixing flavors. The value of having tobacco flavored e-cigarettes was debated, with some saying they would not like tobacco flavors since it would tempt them to smoke but others saying that it helped eased the transition to e-cigarettes. A few participants described unpleasant effects of flavoring (e.g., nausea, throat irritation, burning sensation). Two participants described flavors playing an instrumental role in cigarette smoking cessation. ○ “If I don’t like the flavor, I'm going to smoke a cigarette in a weird way, because it’s not satisfying. It’s like I'm a slave to nicotine, but if you find a flavor that you like, you’re more inclined to be like, “This is sufficient. I don't want (a cigarette).”
Kong, 2015 [30]	E-cigarette	U.S. Adolescents, young adults Users and non-users of e-cigarettes	18 focus groups (127 total participants)	33	Participants reported experimenting with e-cigarettes because of their appealing flavors.
Kotecha, 2016 * [22]	E-cigarette	UK Young adults Users of hookah	16 one-on-one interviews	33	Participants described the flavors of e-shisha as appealing and felt that e-shisha may encourage users to initiate or maintain hookah use. ○ “Non-smokers could then be encouraged to smoke and try tobacco, like actual cigarettes and tobacco flavored shisha.”
Wagoner, 2016 [31]	E-cigarette	U.S. Adolescents, young adults Users and susceptible non-users of novel tobacco products (i.e., electronic cigarettes, hookah, cigarillos, or smokeless tobacco)	10 focus groups (77 total participants)	25	Adolescents and young adults described liking the flavors of e-cigarettes (i.e., cherry, bubble gum, coffee). In differentiating terminology, most participants believed that e-cigarettes did not contain flavors (but did contain nicotine) but that hookah pens did contain flavors (but no nicotine). Participants also described how flavors contributed to the social aspect of using e-cigarettes and were part of the social norm around campus. ○ “Me and my friends will be listening to music in our common rooms and we’ll just be passing it (hookah pen) around and enjoying the smell that the vapor leaves in the air because it smells all fruity and candy-ish and delicious.” ○ “If you are walking around campus, everybody has one (e-hookah). It’s like a grape fruity aroma or sugary aroma following them.”
De Andrade, 2016 [32]	E-cigarette	Scotland Adolescents Users and non-users of e-cigarettes	182 one-on-one interviews	24	Participants described the variety of flavors in e-cigarettes, which led to experimentation. ○ “Loads of people in our school had them. Like, they were walking about and everyone was, like, ‘try this’, because they all had different flavors”. Participants debated whether flavors should be allowed, since some felt that they it would attract younger people to try them. ○ “if they saw like their big brother or something having one and they were like it’s Cola and then the wee bairn (small child) will try it and they will get addicted to them ... that would be how the little one would start smoking, just trying one of them as well ...”
Sterling, 2015 [33]	LCC	U.S. Young adults Users of cigarettes and/or LCCs	12 focus groups (90 total participants)	32	Participants described how varying opinions regarding the harm of flavored LCCs. Some participants thought they were as or more harmful than cigarettes (given their similarity to cigarettes and the presence of warning labels). Others thought they were less harmful because they appeared to be less addictive and because of the flavors and taste, which made them seem more natural. ○ “also the flavors they put the fruits there...people might assume that it’s okay it’s got fruits on it, cause this one’s even got a picture of a fruit all cut up and it looks good. So, they’re associating it with the natural.” “They taste basically like a strawberry. And I like the Tropical Fusion cause it’s like a coconut.” Participants also mentioned experiencing side effects associated with smoking flavored LCCs (i.e., nausea, headaches, dizziness, vomiting, diarrhea).
Sterling, 2015 [34]	LCC	U.S. Young adults Users of cigarettes and/or LCCs	12 focus groups (90 total participants)	32	Participants described the packaging of flavored LCCs positively and noted that smoking flavored LCCs was more tolerable and palatable than smoking unflavored or regular flavor LCCs. ○ “it (flavor) adds to it as opposed to that same bland old tobacco taste. Cigarillo and cigars the tobacco is very harsh on my throat sometimes. But, for some reason flavors just make it a little bit more bearable.” Participants also described choosing to use flavored LCCs to relieve stress or tense feelings. ○ “Yeah, like yellow like the mango ones make me feel like “okay this is going to be a good smoke”. And then the red ones are more like for more of a serious time like after finals. Mango is like a day in the park. And the red one is like after school.”
					Participants reported fruit flavors being for women, whereas man preferred unflavored (or regular) or stronger flavors. ○ “Yeah, women like anything fruity. They (men) like green, black, original, or wine.” Participants also mentioned how flavored LCCs could enhance and accentuate flavored marijuana. ○ “It gives it like an exotic flavor with the weed flavor depending on what grade of weed you’re smoking. If it’s mid or exotic or loud flavor, it accentuates it.” Some participants mentioned how flavors and/or the packaging of flavors led to their experimentation with flavored LCCs. ○ “I had known some people who had already started smoking cigarettes and I’d also been trying cigarillos and you know they were telling me about all the flavors that they had. And that sounded like something that was good to get into.” ○ “and I was like it (LCC) smells good, so it made me want to hit it. But I’m like I don’t like the taste of this. So, then I started of course experimenting with different ones.” Some participants noted that flavoring could affect cigarette smoking cessation. ○ “One of our friends he thought this was a smart idea. He’s like “I’m going to stop smoking cigarettes, like a pack a day. And then I’m just going to do like Black & Milds like BLKs and I’ll just do 2 of them a day because they take longer to burn”. So we thought okay that’s kind of better “cause you’re only doing 2. But at the end of the day, 2 weren’t working out. Like, he had to get more (Black & Milds) throughout the day, so he ended up spending more money. We thought it was a benefit because he was only smoking less, but it ended up not being like that.”
Moodie, 2015 [35]	Cigarettes	Scotland Adolescents, young adults Users and non-users of cigarettes	12 focus groups (75 total participants)	22	In focus groups were participants were shown different colored cigarettes (standard, pink, brown), participants perceived the pink cigarette as less harmful because of its color and indicative of a nicer taste (e.g., strawberry flavored). Participants also noted the appeal of the pink cigarette.
Choi, 2012 [36]	Various	U.S. Young adults Users and susceptible non-users of any tobacco product	11 focus groups (68 total participants)	32	Participants described the flavors in dissolvable tobacco products as fun and interesting. Female participants described the products as “candy that gives you a little buzz”.
Wray, 2012 [37]	Various	U.S. Young adults Users and non-users of tobacco products	8 focus groups (67 total participants)	27	Participants mentioned that products, such as flavored cigarettes or hookah, made people think they were safer than cigarettes. ○ “I think people think it’s safer because there are these other products that are more tolerable for other people than cigarettes are because chewing tobacco, you don’t have the smell of smoke . . . Hookahs and the flavored cigarettes obviously put off a good scent so it’s a lot more tolerable than cigarette use. Participants also mentioned thinking that the industry targeted women and younger people with tobacco flavors.

* study appears twice.
